# Ontology in association rules

**DOI:** 10.1186/2193-1801-2-452

**Published:** 2013-09-11

**Authors:** Inhaúma Neves Ferraz, Ana Cristina Bicharra Garcia

**Affiliations:** ADDLabs – Active Documentation and Design, Instituto de Computação - Universidade Federal Fluminensem, Av. Gen. Milton Tavares de Souza, s/n - 24210-340 Niterói, RJ Brazil; Instituto de Computação -Universidade Federal Fluminense, Av. Gen. Milton Tavares de Souza, s/n - 24210-340 Niterói, RJ Brazil

**Keywords:** Data mining, Association rules, Ontology, Preprocessing, Post-processing, Pruning

## Abstract

Data mining has emerged to address the problem of transforming data into useful knowledge. Although most data mining techniques, such as the use of association rules, may substantially reduce the search effort over large data sets, often, the consequential outcomes surpass the amount of information humanly manageable. On the other hand, important association rules may be overlooked owing to the setting of the support threshold, which is a very subjective metric, but rooted in most data mining techniques. This paper presents a study on the effects, in terms of precision and recall, of using a data preparation technique, called SemPrune, which is built on domain ontology. SemPrune is intended for pre- and post-processing phases of data mining. Identifying generalization/specialization relations, as well as composition/decomposition relations, is the key to successfully applying SemPrune.

## Introduction

New information is created every day and brought to public notice through the Internet or company databases. Data can come from different sources, in different formats and even with different structuring. To gain insights from such large amounts of data, researchers and businesses have developed techniques to manipulate this humanly unmanageable amount of information. In this scenario, data mining has come to be regarded as a useful technique with which to synthesize a large amount of data into more condensed sets of knowledge. Results for various data mining techniques have been promising, when observed at a small scale. There is concrete evidence of the usefulness of data mining of large datasets. Nonetheless, data mining experts are required to navigate and prune the results, looking for useful, unexpected and promising patterns and associations raised in the process (Fayyad et al. 1996; Berry and Linoff 1997; Kantardzic 2003). However, it is not uncommon for data mining to lead to an unmanageable number of outcomes with a great deal of co-related results that only challenge human understanding. Many pre- and post-processing techniques (Jaroszewicz and Simovici 2002; Goethals et al. 2005; Jager 2008) have been proposed to increase the precision and recall of the results. Mostly, these techniques are based on statistical characteristics of the outcome set.

Syntactic proposals of association rule pruning are insufficient because they prune what should not be pruned and refrain from pruning what should. The semantic approach to pruning improves the precision of the results, as filtering does not prune what it should not. Rajan and Dhas (2012) and Nofal and Bani-Ahmad (2010) evaluated and compared favorable results obtained using semantic methods for association rules. Moreover, it has been shown that post-processing can be efficiently integrated with existing rule reduction techniques to construct a concise, high-quality, and user-specific association rule set (Rajan and Dhas 2012; Chen et al. 2008). By integrating objective methods with a semantic approach, it is possible to successfully identify non-trivial, distinctive, semantically correct, and user-specific rules (Chen et al. 2008). There are also semantic-based data mining techniques that consider the taxonomic relations between the elements of the mined results to constrain the outcomes (Silberschatz and Tuzhilin 1996).

Semantic methods improve data mining by bringing known information about the data set to filter and tune the new knowledge extraction. The semantic knowledge can be provided by means of a domain dictionary (taxonomy), a domain ontology in which relationships between concepts are also included, or even a list of expressions that reflect social constructions intended for communication and crystallization of domain-specific knowledge – Sicilia (2006).

Traditional data mining is based on the frequency of occurrence of instances and the co-occurrence of items in transactions. The meaning of each item or instance is not taken into consideration. The semantic content extracted from the ontologies allows inserting more intelligence and knowledge in data mining, improving their quality.

The use of ontologies for best results in data mining has been extensively researched in many respects. Charest and Delisle (2006) developed an ontology-guided method for data mining using case-based reasoning. The method is based on having an expert system assistant to help non-expert data miners (d'Aquin et al. 2012).

Žáková et al. (2008) use a domain ontology and a task description to create a workflow for guiding data mining process. The knowledge discovery task is converted in a planning task.

Manda et al. (2013) presented a data mining approach, entitled multi-ontology data mining at All Levels (MOAL), that uses the structure and relationships of a Genetic Ontology to mine multi-multi-level association rules.

Zhou and Geller (2008) use ontologies to enrich the web mining for marketing domain. Their work uses two taxonomies (customers and interests) in the data mining pre-processing phase to improve the precision and recall of the rules. Their proposal is the very similar to the SemPrune model presented in this paper.

The purpose of the SemPrune is to generate better data mining results on database for any task or domain through ontology-enrichment of data. Generalization and specialization information guide merging, deleting or adding knowledge rules.

The idea behind SemPrune comes from observations of ecommerce transactions. Consider a dataset of online sales transactions. The simplest way of using subjacent knowledge is to group attributes into value ranges such as by grouping purchasable items by price range (cheap, normal price, expensive), individuals by height (short, medium, tall), or workers by productivity (low, medium, high, excellent). Comprehensive variables, such as the price range of items, generalizers, or grouping representatives, are those which, according to the information-storage perspective, are called dependent, since whenever a specific (or determinant) variable is known, the comprehensive (or dependent) variable is also known. Each type of relationship has its representation and resolution dependency mechanisms. Usually the determinant attributes (which give rise to aggregating attributes) occur in small numbers. This type of analysis is often used in the case of value ranges of attributes and derived aggregator attributes. A pre-processing activity would normally create new rules with the dependent variables established in a file of dependencies. The present research applies the semantic enhancement of rule mining along with the pruning treatment.

Aggregating variables can provide more general or comprehensive rules. These rules can be compared to the mined rules through metrical structures based on objective measurements (coverage in post-processing or distribution uniformity in pre-processing) that indicate if the new rule may substitute a set of original rules (generalization) or if it does not add information, in which case it must be unconsidered (rule specialization). The comprehensive rules derive from domain ontology. The treatment of the set of mined rules employing filtering techniques for the reduction of cardinality is a classic treatment, and the innovation presented in this paper is semantic filtering in lieu of syntactic filtering employing objective rules of interest.

We proposed an ontology-based model, called SemPrune (Ferraz 2008), which combines a semantic pre-processing of the data set with a semantic post-processing of the data mining outcomes. We claim that making use of known domain concepts and relations can positively affect data mining recall and precision, while reducing the volume of the outcomes. We have developed an experiment using public data sets (Lucas et al. 2002; Merz and Murphy 1998; Collective Bargaining Review, monthly publication 1998), the association rules’ data mining technique and domain ontology related to the datasets. Experiments, using SemPrune, have given results that are more concise and precise than the results obtained using semantic filtering techniques, such as Conviction, Specificity, Lift and Novelty (Lavrac et al. 1999). The comparison was performed quantitatively and qualitatively. The quantitative comparison was performed according to the number of pruned rules (recall). The qualitative comparison was performed by human evaluation.

In this paper, we describe our research problem, and then SemPrune. Experiments and a comparison of our approach with other research works are presented.

## Background

### Ontology

According to Gruber (1993), ontology is a conceptualization of a specification. Instead of using this very broad explanation, the present research takes a more tangible definition of ontology as a domain representation in which the concepts and the relations among them become explicit to allow either human negotiation of the denotations and/or machine inferences for a specific application. People build domain ontology, and consequently, rarely is there a consensual understanding of all concepts, and different ontologies can be built to describe the same domain. Since the 1990s, there have be have been many studies not only on techniques with which to build (Musen 1992; Protégé^2000^) and map ontologies (Euzenat and Shvaiko 2007), but also on applications that could benefit from representing knowledge using domain ontology, such as knowledge-based systems, information retrieval mechanisms, agent communication language definition and search methods. The present research investigates the effects of using domain ontology to improve the precision and recall of association-rule extraction from large databases.

Identifying the concepts and relations between concepts is crucial to building an effective domain ontology. Although the semantics of the relations can be defined as one builds an ontology, there are some relations with established meanings, such as “is-a”, “part-of” and “attribute-of”. The first relation, is-a, comes from the set theory relation set and subset. Consequently, the subset carries all definitions of the set. If Peter is-a human, then Peter inherits all characteristics of being a human. This description can be very useful in describing concepts in a more concise way.

The second relation, part-of, brings to bear the concept of composition/decomposition. The parts make the whole. A motor engine and chassis are parts of a car. A motor alone cannot be considered a car.

Attribute-of is a simple relation that represents properties of the concepts. For example, a car has a color and might have an owner. Both are attributes of a car. It is natural to map the way one describes a domain in natural language and the relations the concepts present. However, care needs to be taken for language might be misleading. A car has a color and has a motor. Although, in general, we use the same verb to connect the car and color, and the car and motor engine, the relations between these concepts are different. In the first one, the relation is clearly about characteristics of the object, while in the second one, the relation refers to composition.

### Association rules

The use of association rules is a popular technique of mining data; the technique shows the correlation between sets of items in a series of data or transactions. Association rules are an “IF antecedent THEN consequent” type of rule that guarantees, with a certain probability (confidence threshold), that whenever the antecedent happens, the consequent will follow. These rules are generated from sets of elements (itemsets) that appear together with at least some frequency (support). The most popular algorithm for obtaining association rules is Agrawal’s apriori (Agrawal and Srikant 1994). Considering a fixed confidence value, the setting of the support threshold will determine whether too many association rules are set or important relationships in the data are missed.

#### Semantic treatment of association rules

Pairs of association rules for which items in the antecedent are semantically correlated can be simplified as one single association rule, either more comprehensive or more specific, depending on the context.

In generalizations, we value the summarized view of discovered relationships, whereas in specializations, we value the rules’ discriminatory ability. To verify whether general rules can substitute various specific rules, one must check if the general rules provide enough coverage over all the specific rules. In this case, the rules with instances in the antecedent can be pruned. If not, there are singular specific rules that do not fit into the general rule and cannot be pruned.

This semantic treatment of using predefined domain knowledge to prune an association-rule outcome can be used during post- and pre-processing as described below.

#### Semantic post-processing

Our semantic post-processing consists of, initially, enhancing the rule with domain information for later analysis and decision upon pruning. Each more general enhancing rule should be able to substitute a number of specific rules by way of a generalization process. If this is possible, there are simultaneously a semantic enhancement of the set of mined association rules and a future reduction in the cardinality of the set of rules.

Our post-processing technique does not work if the dependent attribute chosen in the ontology selection doesn’t cover the determinant attributes. Whenever the results to be added do not have a reasonably coverage, the specialization will discard the aggregating rule that was semantically generated.

The CRg indicator, defined in formula 1, measures the generality of the more general rule in relation to the attributes that determined the dependent variable. This measure is based on the Coverage interest measure (Lavrac et al. 1999), which represents the fraction of instances covered by the antecedent of the rule. This can be considered a measure of rule generality. The value of the Coverage of a rule is given by the support of the antecedent of this rule.

**Definition***(CRg). Let D be a multidimensional database. Let R*_i_*be an association rule in the form* Y_i_ ^ A ⇒ B*, and the set of corresponding rules in the form* X_ij_ ^ A ⇒ B*, obtained from D. The value of the measure CRg for R*_i_*and S*_i_*is given by*1

The larger the measure of CRg, the larger the representability of the instances covered by the more specific rules in relation to those instances covered by the more general rule, which in turn means a uniform behavior of the population. The measure CRg can be interpreted as the conditional probability that an instance could satisfy the antecedent of one of the more specific rules, given that the instance satisfies the antecedent of the more general rule.

#### Semantic pre-processing

The choice between generalization (preferred) and specialization can be made by assuming a uniform distribution of support of rules containing dependent attributes that have a common father. The natural solution would be to use statistical rules to obtain the values outside of the "mean" range (i.e., "outliers"). It happens that the standard search for outliers is to look for values well above and well below expectations. In the case presented, the specific association rules with low support have to be pruned. Usually, the characterization of outliers is made for deviation greater than 1.5 or 2.0 standard deviations of the distribution. Experimentally, we determine a minimum standard deviation to consider the meaninglessness of outliers.

With the values obtained, we calculated the support standard deviation of the distribution of specific rules. If the ratio of the standard deviation and arithmetic mean of the distribution is below a specified threshold, the behavior of specific rules is regular, and these rules may be replaced by the general rule (and be pruned). Otherwise, there are singular specific rules that do not fit the general rule and cannot be pruned.

**Definition** (TRg). *Let D be a multidimensional database. Let R*_*i*_*be an association rule in the form Y*_*i*_*^ A ⇒ B, and S*_*i*_ = {*r*_*ij*_|*j* = 1.. *n*} *a set of corresponding rules in the form X*_*ij*_*^ A ⇒ B, obtained from D. Let x*_*k*_*be the value of the support of a rule r*_*ij*_*and μ the value of the arithmetic mean of the support of these rules. Let σ be the standard deviation support of the population of rules. The value of the measure TRg (formula 2) for R*_*i*_*and S*_*i*_*is given by the inverse of the coefficient of variation.*2

Where

The greater the value of TRg, the less the average deviation of the support of specific rules in relation to the average, meaning the small relative importance of specific rules with high support (singular rules). When the distances of the support of rules are greater than the minimum threshold for consideration of the regular uniformity of the population (TrgMin), singular rules will be considered those rules for which the distance of their support relative to the average of supports of the distribution divided by the population standard deviation is greater than α × TRgMin (where α is an empirical coefficient that is a characteristic of the domain); i.e., those rules that respect the inequality.3

The subsets of rules that present the same consequent are generated by the algorithm described by Jager (2008). For each of these subsets, a set G of more general rules is generated. In a second stage, each general rule generated is analyzed to verify its generalization capacity.

The next step begins by generating the set E of more specific rules that are redundant with each general rule. According to the set of more specific rules, the value of measure TRg is calculated. If the value of the TRg measure is greater or equal to the minimum value specified by the user (TRgMin), the more specific rules are eliminated by way of a generalization process. If it is not, the process that is executed is the rule specialization with the elimination of the more general rule.

## SemPrune model

The SemPrune model is presented in two versions that are composed of a number of modules. The descriptions of the algorithms in these modules are condensed as much as possible to meet the specifications of the Conference, but the text in full is available upon request.

When SemPrune makes the semantic enrichment,we find a more general rule and several specific rules. In the preprocessing enrichment, the computed value of CRg is compared with the specified CRgmin value. When CRg ≥ CRgmin the specific rules can be discarded because they are represented by the more general rule. Otherwise, the discarded rule is the more general rule. In tests with CRgmin it was found that the best results for this threshold varied between different databases (from 0.5 to 0.8). For enrichment in preprocessing the similar comparison was made between TRg and TRgmin.

### SemPrune for post-processing

**Figure 1:**
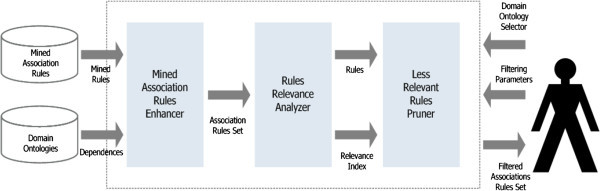
**Post-processing semantic enrichment model.**

The SemPrune version for enriching the post-processing is illustrated in Figure 1. The Transactions’ database is the source for the data mining technique to extract a set of association rules. The rules obtained from this and the domain ontology are then sent to an enhancer module that enhances the set of association rules with dependent attributes that provide a higher power of intuition for the analyst. From this point forward, there is a confluence between the two versions.

The redundancies between rules were analyzed according to the premise that there are always dependencies between attributes and rules, because some rules may have, in the antecedent or consequent, determinant attributes and determined attributes. The redundancy between them can be of four types:

- the antecedent of the rule has determinant attributes and the consequent has a dependent attribute (redundant with the premise) of the form "If there is a rule of the form X ⇒ Y ^ C, this is redundant, and C is a set of conditions defined on attributes of D";
- a rule has, in the antecedent, determinant attributes, and another with the same consequent, has, in the antecedent, determinant attributes plus the dependent attribute (second rule is redundant with the first) of the form "If there are two forms of rules R1: X ^ A ⇒ B and R2: X ^ Y ^ A ⇒ B, then rule R2 is redundant, A and B being defined sets of conditions on attributes of D;
- a rule has, in the antecedent, determinant attributes, and another, in the consequent, the dependent attribute plus the consequent of the first rule (the second rule is redundant with the first) of the form "If there are two forms of rules R1: X ^ A ⇒ B and R2: X ^ A ⇒ Y ^ B, then rule R2 is redundant”;
- a rule has, as part of the antecedent, a dependent attribute, and another rule has as a sole difference the replacement of the dependent attribute by their determinant attributes (second rule is redundant with the first) of the form "If there are two forms of rules R1: Y ^ A ⇒ B and R2: X ^ A ⇒ B, then rule R2 is redundant".

### SemPrune for pre-processing

**Figure 2:**
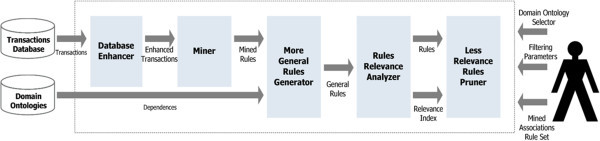
**SemPrune’s semantic enrichment during the data mining pre-processing phase.**

The pre-processing semantic enrichment SemPrune version is illustrated in Figure 2. Based on a transactions database and a domain ontology of the items of the transactions, a database enhancer module generates an enriched set of transactions. This transaction set is mined to generate an enhanced set of mined association rules. This set of rules and the domain ontology feed a generator module that, for every more general rule, it locates a set of corresponding specific rules. These sets of rules (for each more general rule there is a subset of specific rules) are analyzed by a rules relevance analyzer module, whose output is made up of the entrance sets with the corresponding relevance indexes. Finally, a less relevant rules pruner module executes a filter whereby only the semantically more relevant rules of the sets of rules (more general + specific) will succeed.

The proposed method for the problem solution then aims to eliminate redundant association rules, or less impact rules based on the dependency relationships between attributes, assuming that the information enrichment has already been done in the preprocessing of association rules mining.

The method adopted to solve the problem is similar to that used for filtering with the inclusion of world knowledge in the post-processing. The biggest difference is the definition of dependence between attributes. In the case of a relationship 1:n, it is considered that the descendent in the taxonomy is determinant of its ancestor, which implies greater simplicity in the calculation of values of dependent attributes for grouping, separation, comparison, and so on. In contrast, the number of dependencies increases, because within the hierarchy, every attribute has a father (just has dependency).

With the values obtained, we calculate the standard deviation of the support distribution of specific rules. If the ratio of the standard deviation and arithmetic mean of the distribution is below a specified level, the behavior of specific rules is regular, and they can be replaced by the general rule and pruned. Otherwise, there are singular specific rules, which do not fit the general rule and cannot be pruned. We must then determine if the general rule is representative of non-singular specific rules. If the general rule covers sufficiently the non-singular specific rules, the latter will be pruned. Otherwise, it is the general rule that brings no value, and can be pruned.

### Experimental evaluation

To evaluate the results obtained from the semantic enhancements made in the post-processing phase, we used databases from usual Internet repositories and introduced dependent attributes, like the range and aggregator, which is common practice in data mining. In each case, the rule enhancements were compared using knowledge of the world during pre-processing and post-processing. The association rules were extracted using the apriori algorithm (Lucas et al. 2002). Domain ontology emphasized “is-a” and “part-of” relations among domain concepts. We selected the adult database (32,561 records) and labor database (57 records) publicly available from the UCI Machine Learning Repository (Merz and Murphy 1998). The STULONG database (1317 records) was obtained from ECML/PKDD 2004 Discovery Challenge (ECML/PKDD, 2004).

World knowledge aggregated to mined data will generate redundancy, since wide-ranging attributes and specific attributes will coexist. Wide-ranging attributes, such as price range of items, pattern generalizers, or grouping representatives, are called dependents under the information storage point of view, since once a specific (or determinant) attribute is known, the wide-ranging (or dependent) attribute is also known.

For the adult database we used 14 attributes of the data base and from the World Knowledge we included three dependent attributes (Hollingshead Index of Social Position - ISP, social-class and Age-group). The target attribute was the income. For the stulong database we used 18 attributes of the data base and from the World Knowledge we included three dependent attributes (Body Mass Index - BMI, Status and Age-group). The target attribute was the blood-pressure. For the labor database we used 16 attributes of the data base and from the World Knowledge we included two dependent attributes (Wage-inc and Sweat-hours). The target attribute was class.

The support values adopted were 4% for the labor database and 2% for the other two databases. The confidence value adopted in all mining cases was 70%. The maximum size specified for the frequent itemsets was three for the STULONG database and five for the other two databases.

Northwind Traders is a database of a fictitious trading company, provided as an example, with Microsoft products Access and SQL Server, the purpose of which is to support applications and MBP ("Modeling Business Processes").

To turn it into a database transaction, which allows data mining of user behavior, it was necessary to create an adequate view. Data on the purchasing habits of users are recorded in Order Details, Customers and Orders tables.

The attributes that are part of the vision of database transactions were CustomerID, and ProductID Order Date, and that will be part of world knowledge are ProductID and CategoryID.

**Figure 3:**
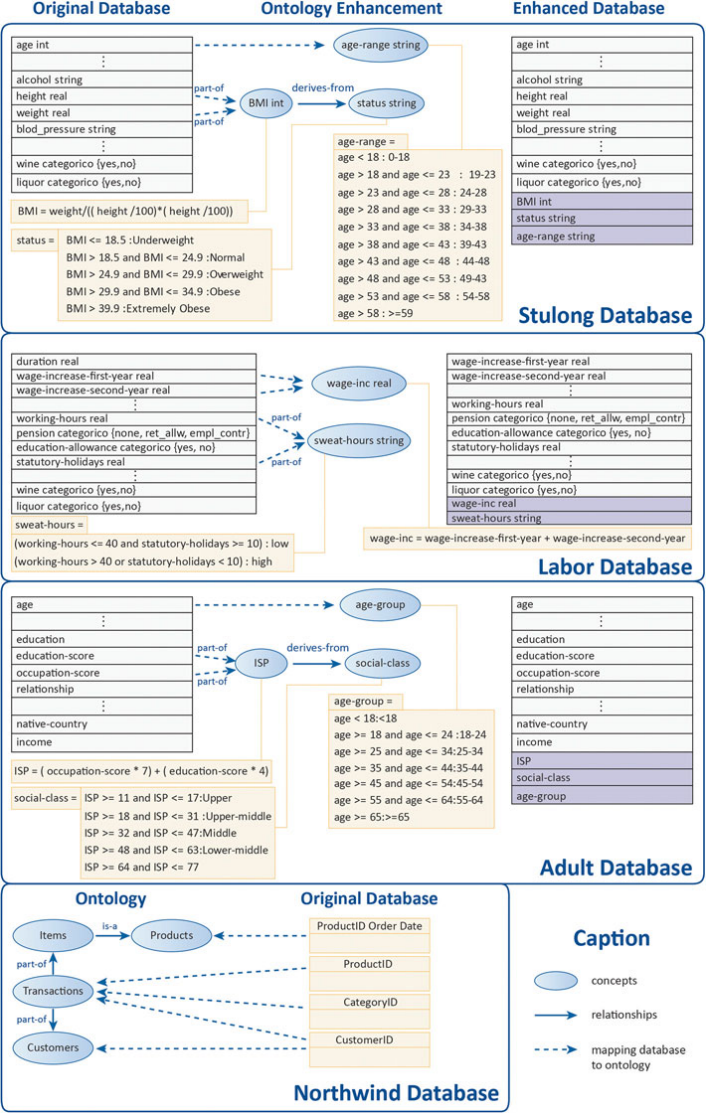
**Partial view databases and ontologies interaction.**

The concepts and relationships of the ontologies used in this work can be seen in Figure 3.

The support values adopted were 4% for the labor database and 2% for the other two databases. The confidence value adopted in all mining cases was 70%. The maximum size specified for the frequent itemsets was three for the STULONG database and five for the other two databases.

## Results and discussion

Table 1 is a summary of the results obtained using the proposed method on the three selected databases. The first column of the table gives the database, the second column the number of association rules mined, the third column the number of rules with dependency, the fourth column the number of rules considered redundant and eliminated by the semantic method, and the fifth column the ratio between the number of rules eliminated and the total number of rules mined.

**Table 1:** **Effect of SemPrune post-processing on the number of extracted rules**

Database	Mined rules	Rules with dependency	Eliminated rules	Reduction percentage
Adult	2924	1988	515	17.61%
Stulong	34422	12601	8163	23.71%
Labor	181229	134962	32874	18.14%

It is observed that the semantic filter was effective for all sample databases. The reduction rates were reasonable, reaching more than 23% for the STULONG database. The proposed semantic method decreases the number of rules in addition to providing more consistency to the results in each case.

To test the effectiveness of SemPrune in the data pre-processing phase, we used the Northwind Traders database. This is a database of a fictitious trading company, shipped as a sample with Access and SQL Server Microsoft products, whose purpose is to support database applications and modeling business processes. The database consists of eight main tables and seven auxiliary tables, and comes with 16 views and seven stored procedures.

The transactions database was generated by the junction of the Orders and OrderDetails tables, creating itemsets of products purchased by the same CostumerID. The world knowledge, the base for the taxonomic relationship, was obtained by joining the Categories and Products tables.

For relationships with the inclusion of world knowledge in the pre-processing, the generated rules had a maximum of five items, minimal support of 0.04, and 9941 mined rules for the Northwind Traders database.

Applying the SemPrune method to the pre-processing of the Northwind Traders database, we obtain the results presented in Table 2. The first column gives the database, the second column the number of association rules mined, the third column the number of rules considered redundant and eliminated by the semantic method, and the fourth column the ratio between the number of rules eliminated and the total number of rules mined.

**Table 2:** **Effect of SemPrune pre-processing on the number of extracted rules**

Database	Mined rules	Eliminated rules	Reduction percentage
Northwind Traders	9941	5052	50.82%

Table 3 shows the gain in precision obtained by applying the SemPrune model knowledge in post-processing. In all the databases used, no rule was erroneously pruned and the amount of information recovered decreased. The gains ranged from 21% (adult database) to 31% (STULONG database). The recall remained constant since neither the intersection of the sets of relevant information and the information retrieved nor the relevant information set changed.

**Table 3:** **Effects of SemPrune on precision**

Database	Mined rules	Eliminated rules	Remaining rules	Precision gain
Adult	**2924**	**515**	**2409**	**21.38%**
Stulong	34422	8163	26259	31.09%
Labor	181229	32874	148355	22.16%

The superiority of semantic filtering can be verified by comparing results thus generated with post-processing filtering guided by objective interest measures, specifically Conviction, Specificity, Lift and Novelty (Lavrac et al. 1999).

The four objective measures were applied using five different values for each objective interest measure, as the cutoff point. Table 4 shows the result obtained for the STULONG database. As shown, the objective filters are lenient, with redundant rules being detected by semantic filters. It is confirmed in this experiment that the objective filters have a set too sensitive so that small variations in the cutoff produce significantly different results. Such behavior requires a lot of expertise of users, which generally leads to poor quality of the result. Taking the example of the STULONG database, it appears that a variation of only 0.02 at a cutoff value of specificity (0.95 to 0.97) caused the number of rules left to drop from 2583 to 1307, a reduction of 49%. A variation of only 0.10 for a cutoff value of lift (1.00 to 1.10) caused the number of rules left to drop from 2950 to 921, a reduction of 68%.

**Table 4:** **Results obtained using the interest measures for the STULONG database**

Conviction	Cut-off	1.1	1.2	1.3	1.4	1.5
	% Eliminated Rules	48,85%	53,33%	58,04%	61,45%	63,18%
	# Remaining Rules	1597	1377	1238	1137	1086
	SemPrune % of Eliminated rules	26,35%	16,26%	14,47%	12,60%	11,54%
Specificity	Cut-off	0.95	0.97	0.98	0.99	1.00
	% Eliminated Rules	12,44%	26,33%	5,22%	53,43%	59,38%
	# Remaining Rules	2583	1307	2180	1374	1198
	SemPrune % of Eliminated rules	12,83%	8,91%	2,46%	0,65%	0,00%
Lift	Cut-off	1.0	1.1	1.2	1.3	1.4
	% Eliminated Rules	0,00%	20,86%	24,89%	25,30%	26,06%
	# Remaining Rules	2950	921	2216	2204	2181
	SemPrune % of Eliminated rules	17,61%	5,84%	0,00%	0,00%	0,00%
Novelty	Cut-off	0.0	0.1	0.2	0.3	0.4
	% Eliminated Rules	0,00%	10,00%	100,00%	100,00%	100,00%
	# Remaining Rules	2950	0	0	0	0
	SemPrune % of Eliminated rules	17,61%	0,00%	0,00%	0,00%	0,00%

For the qualitative comparison of data mining post-processing between syntactical methods (pruning using Conviction, Specificity, Lift and Novelty) and semantic methods (pruning using generalization/specialization), performance tables can be employed. The qualitative comparison must be conducted in light of the domain ontology through the observation of an analyst who can attest to the conformity of the results obtained by filtering with the domain ontology.

In the performance table, shown in Table 5, similar to the case for confusion matrixes, it can be said that the pruning of any rule can be viewed in light of two aspects:

- if it should (S) or should not (SN) have been pruned, as analyzed from a semantic point of view and shown in the rows of the table;
- if it was (W) or was not (WN) pruned, as attested by the application of the filtering via an objective measure of interest and shown in the columns of the table.

**Table 5:** **Performance metrics for the STULONG database**

Conviction				Specificity				Lift				Novelty			
Cut-off		W	WN	Cut-off		W	WN	Cut-off		W	WN	Cut-off		W	WN
1.1	S	807	966	0.95	S	212	1561	1.0	S	0	1773	0.0	S	0	1773
	SN	546	631		SN	155	1022		SN	0	1177		SN	0	1177
1.2	S	923	850	0.97	S	466	1307	1.1	S	359	1414	0.1	S	1773	0
	SN	650	527		SN	311	866		SN	256	921		SN	1177	0
1.3	S	1004	769	0.98	S	72	1051	1.2	S	429	1344	0.2	S	1773	0
	SN	708	469		SN	48	1129		SN	305	872		SN	1177	0
1.4	S	1053	720	0.99	S	943	830	1.3	S	436	1337	0.3	S	1773	0
	SN	760	417		SN	633	544		SN	310	867		SN	1177	0
1.5	S	1079	694	1.00	S	1048	725	1.4	S	449	1324	0.4	S	1773	0
	SN	785	392		SN	704	473		SN	320	857		SN	1177	0

The main diagonal of Table 5 presents the number of rules that should and were pruned and the number of rules that should not and were not pruned. These values indicate the degree to which the syntactical methods were correct. The secondary diagonal of the tables presents the number of cases in which the measure of objective interest did not function correctly, eliminating rules that should not have been kept and keeping rules that should have been eliminated. For example with conviction of 1.5, we see that the main diagonal sum (1079 + 392) is greater than the secondary diagonal sum (785 + 694).

For relationships with the inclusion of world knowledge in the pre-processing, the generated rules had a maximum of five items, minimal support of 0.04, and 9941 mined rules for the Northwind Traders database. The superiority of the results obtained by semantic filtering in the pre-processing can thus be seen by comparing results generated by filtering according to objective interest measures, specifically Conviction, Specificity, Lift and Novelty (Lavrac et al. 1999).

The four objective measures were applied using five different values for each objective interest measure, as the cutoff point. Table 6 presents the results. The table shows that objective filters are lenient, with redundant rules being detected by semantic filters. It is confirmed in this experiment that the objective filters have a very sensitive adjustment so that small variations in the cutoff produce significantly different results. Such behavior requires much user expertise, which usually leads to low quality of the result. A variation of only 0.02 in the cutoff of Specificity (from 0.97 to 0.99) resulted in a drop from 1389 to 892 (down 36%) in the number of eliminated rules and an increase of 0.10 (from 0.00 to 0.10) in Novelty resulted in a decrease from 5294 to 97 in the number of eliminated rules.

**Table 6:** **Interest measures for the Northwind traders database**

Conviction	Cut-off	1.1	1.2	1.3	1.4	1.5
	% Eliminated Rules	16,50%	16,50%	16,50%	16,79%	17,05%
	# Remaining Rules	4515	4515	4515	4485	4499
	SemPrune % of Eliminated rules	83,01%	83,01%	83,01%	83,03%	83,04%
Specificity	Cut-off	0.95	0.97	0.98	0.99	1.00
	% Eliminated Rules	59,20%	74,31%	77,07%	83,50%	83,50%
	# Remaining Rules	2206	1389	1240	892	892
	SemPrune % of Eliminated rules	84,27%	80,49%	71,37%	69,06%	62,89%
Lift	Cut-off	1.0	1.1	1.2	1.3	1.4
	% Eliminated Rules	0,00%	0,00%	0,00%	0,00%	0,55%
	# Remaining Rules	5407	5407	5407	5407	5377
	SemPrune % of Eliminated rules	79,67%	79,67%	79,67%	79,67%	79,69%
Novelty	Cut-off	0.0	0.1	0.2	0.3	0.4
	% Eliminated Rules	0,00%	98,17%	99,93%	100,00%	100,00%
	# Remaining Rules	5407	0	0	0	0
	SemPrune % of Eliminated rules	76,67%	100,00%	100,00%	0,00%	0,00%

The performance matrices with the inclusion of world knowledge in the pre-processing are shown in Table 7. The table shows the difference of the semantic proposal object of the present research.

**Table 7:** **Performance table for the Northwind traders database**

Conviction				Specificity				Lift				Novelty			
Cut-off		W	WN	Cut-off		W	WN	Cut-off		W	WN	Cut-off		W	WN
1.1	S	561	3747	0.95	S	2671	1637	1.0	S	0	4308	0.0	S	0	4308
	SN	331	768		SN	530	569		SN	0	1099		SN	0	1099
1.2	S	561	3747	0.97	S	3353	955	1.1	S	0	4308	0.1	S	4235	73
	SN	331	768		SN	665	434		SN	0	1099		SN	1073	26
1.3	S	561	3747	0.98	S	3454	854	1.2	S	0	4308	0.2	S	4304	4
	SN	331	768		SN	713	386		SN	0	1099		SN	1099	0
1.4	S	572	3736	0.99	S	3747	561	1.3	S	0	4308	0.3	S	4308	0
	SN	336	763		SN	768	331		SN	0	1099		SN	1099	0
1.5	S	584	3724	1.00	S	3747	561	1.4	S	23	4285	0.4	S	4308	0
	SN	338	761		SN	768	331		SN	7	1092		SN	1099	0

The evaluation of the effect of applying the SemPrune model with the inclusion of world knowledge in pre-processing is presented in Table 8. The table gives the number of rules originally mined, the number of enriched rules obtained by applying the SemPrune model, the number of rules eliminated by the model, the number of remaining rules and the recall gain. The inclusion of world knowledge greatly increased the number of rules retrieved. If there was no difference in the number of relevant rules, any gains would arise from the expansion of the numerator. In any event, no matter how small the fraction of relevant rules included in the SemPrune model, it will be greater than zero, and consequently, the gain in recall is not zero.

**Table 8:** **SemPrune and recall**

Database	Number of mined rules	Number of rules inthe enriched rules’ set	Number of eliminated rules	Number of remaining rules	Recall gain
Northwind Traders	6070	9941	5052	4889	63.77%

However, this gain cannot be generalized, as it depends on the ontology. A high number of attributes in the database that have ancestors increases the probability of pruning. (The number was too high in the case of the Northwind Traders database.) The alternative hypothesis specified only the recall gain and this has been achieved.

To assess the effect of SemPrune model, when the inclusion of world knowledge occur during post-processing, is set to Table 9 presents the results of using SemPrune applied to three different databases.. As shown in Table 9, there was no significant difference in the results considering the number of relevant retrieved rules. For the Adult database, the gain was 21.38%.

**Table 9:** **Precision gain of SemPrune model**

Database	Mined rules	Eliminated rules	Remaining rules	Precision gain
Adult	2924	515	2409	21.38%
Stulong	34422	8163	26259	31.09%
Labor	181229	32874	148355	22.16%

The highest effects of applying the SemPrune occurs in pre-processing phase, as presented in Table 10. The table shows the number of rules originally mined, the number of enriched rules obtained by applying the SemPrune model, the number of rules eliminated by the model, the number of remaining rules and the recall gain. Assuming that all rules obtained by the semantic enrichment are relevant, the result would be 100% ( # Enriched rules set / # Mined rules ). The actual determination of the amount of relevant rules revealed by the domain ontology can only be done by human inspection. Anyway, no matter how small the fraction of relevant rules included by SemPrune model, it will be greater than zero and, consequently, the gain in "recall" is not null. The accuracy, calculated by the ratio between the number of retrieved relevant rules and the number of recovered rules, grows as the number of remaining rules is less than the original rules. However, this gain cannot be generalized because it depends on the ontology.

**Table 10:** **Recall gain of SemPrune model**

Database	Mined rules	Enriched rules set	Eliminated rules	Remaining rules	Recall gain
Northwind Traders	6070	9941	5052	4889	63,77%

## Conclusions

The study described in this article demonstrated the positive effect of using domain ontology to prune association-rule outcomes. We addressed the issue of excessive, though redundant, association rules being generated by traditional data mining techniques. The result, which we were searching for, was a reduction of the set of mined rules while maintaining precision and reducing recall. Related work was carried out by Srikant and Agrawal (1995) and Bürkle (2006). In the first work, the filtering was conducted by a frequency model (interestingness), and in the latter, associations rules were enhanced in the pre-processing step and only for “is-a” relationships.

Recall is already efficiently reduced by syntactic filtering measures that use objective interest measures of the rules. Nevertheless, the fact that there are several dozen competing objective measures is already an indication that none of them is absolute. When analyzing the pruned rules using these various measures, it becomes clear that the degree of superposition is low, and therefore, a loss in precision is inevitable.

SemPrune is limited to datasets for which domain ontology is available and for which data items are considered balanced. Since the technique uses intrinsic semantic relations between values of the same attribute, imprecise domain description may drastically affect the results. Additionally, whenever the dataset is unbalanced, there is a risk of false generalization. For example, if the data set only contains data including Coke and Pepsi, SemPrune might lead to generalized rules of non-alcoholic beverages because there is no evidence in the data set of any other drink.

Our suggestion, which uses knowledge of the world obtained from domain ontology, may enhance the semantics of the rule set obtained and substantially reduce its cardinality. Furthermore, semantic rule pruning does not eliminate relevant rules nor does it fail to eliminate redundant rules.

The results of the experiments obtained with public databases show that the suggested model fully met the desired goals. As a side effect, the integration passage of the pre-processing ontology to the post-processing significantly reduces computational costs, as shown by the number of rules to treat.

SemPrune is based on the classic way humans learn by anchoring new knowledge on previous knowledge, finding shortcuts to generalize, and identifying scenarios for which specifics matter. Our results are cautious steps towards merging these two powerful areas of data mining and domain ontology representation to face the challenge of dealing with big data.

## Authors’ information

Inhauma Neves Ferraz was born in Brazil, in 1940. He received a B.E degree in civil engineering from Instituto Militar de Engenharia, and a B.S. degree in mathematics from Universidade Federal do Rio de Janeiro. He obtained M. Sc. degrees in mechanical engineering from Universidade Federal de Itajubá and in system engineering from Instituto Militar de Engenharia. A D. Sc. Degree in computer science was obtained from Universidade Federal Fluminense.

He has held lecturing positions at the Universidade Estadual Paulista Júlio de Mesquita Filho, Universidade Federal de Itajubá, Instituo de Tecnologia de Aeronáutica, Instituto Militar de Engenharia e Universidade Federal Fluminense.

His main areas of research interests are information security, artificial intelligence and computer networks.

He is currently Vice Director of ADDLabs, the Artificial Intelligence laboratory of Computer Science Institute at Universidade Federal Fluminense.

He has published more than 40 scientific papers, a book and more than 30 technical reports on computer science.

Ana Cristina Bicharra Garcia is a professor at the Computer Science Department of the Universidade Federal Fluminense, Brazil. She created ADDlabs in 1996 and has since directed the laboratory; ADDlabs is an artificial intelligence research laboratory focused on the petroleum exploration domain (http://www.addlabs.uff.br). She received her M.Sc. (1988) and Ph.D. (1992) degrees from Stanford University. She has advised seven PhD students and approximately 30 MSc students. She has written numerous papers on artificial intelligence applied to design, data mining techniques, cooperative work, e-government, e-commerce and knowledge acquisition. Her current interests involve knowledge acquisition and collective intelligence. She has published 20 journal papers, 12 book chapters and 124 conference papers.
